# Bildgebung von Lebertumoren im Kindes- und Jugendalter

**DOI:** 10.1007/s00117-021-00851-1

**Published:** 2021-06-01

**Authors:** S. Tschauner, M. Riccabona

**Affiliations:** Klinische Abteilung für Kinderradiologie, Universitätsklinik für Radiologie, Medizinische Universität Graz, Auenbruggerplatz 34, 8036 Graz, Österreich

**Keywords:** Leberläsionen, Staging, Bildgebung, Sonographie, Magnetresonanztomographie, Liver neoplasms, Staging, Imaging, Sonography, Magnetic resonance imaging

## Abstract

**Hintergrund:**

Kindliche Lebertumoren sind relativ selten, aber die Kenntnisse über ihre bildgebende Diagnostik nicht weniger wichtig.

**Fragestellung:**

Häufigkeit und Bildmorphologie benigner und maligner Raumforderungen der Leber im Kindesalter.

**Material und Methode:**

Aufbereitung der einschlägigen Originalarbeiten, Übersichtsarbeiten und Expertenempfehlungen betreffend die Bildgebung kindlicher Lebertumoren.

**Ergebnisse:**

Die häufigsten bösartigen Lebertumoren sind das meist bei Kleinkindern auftretende Hepatoblastom sowie in manchen Regionen auch das hepatozelluläre Karzinom. Ihre Bildmorphologie ist im Gegensatz zu manchen benignen Raumforderungen, wie beispielsweise der fokalen nodulären Hyperplasie, simplen Zysten oder Steatosearealen, wenig charakteristisch. Radiologisch kommen üblicherweise die Sonographie und die Magnetresonanztomographie (MRT) zum Einsatz. Beide Methoden profitieren von einer intravenösen Kontrastmittelgabe.

**Schlussfolgerung:**

Kindliche Lebertumoren weisen ein breites morphologisches Spektrum auf. Manche Entitäten lassen sich mittels Bildgebung charakterisieren, manche benötigen eine multimodale Bildgebung oder eine histologische Begutachtung. Neben den bildmorphologischen Kriterien spielen insbesondere auch Alter, Laborparameter und Anamnese eine wichtige Rolle in der Diagnosefindung.

## Abstract (en)

**Background:**

Pediatric liver tumors are relatively rare, but thorough knowledge of their imaging features is still important.

**Objectives:**

Frequency and imaging features of benign and malignant liver masses during childhood and adolescence.

**Materials and methods:**

Discussion of relevant original articles, review manuscripts and expert recommendations concerning imaging of childhood liver tumors.

**Results:**

The most common malignant tumors of the liver are hepatoblastoma, which usually occur in younger children, as well as in some regions hepatocellular carcinoma. In contrast to most benign masses, such as focal nodular hyperplasia, simple cysts or fatty liver infiltrations, their imaging morphology is not very characteristic. Radiologically, sonography and magnetic resonance imaging (MRI) are used for assessment. Both methods benefit from intravenous contrast agent administration.

**Conclusions:**

Childhood liver tumors show a broad spectrum of morphological manifestations. Some entities can be characterized using standard imaging, some require multimodal imaging or histological assessment. In addition to morphological imaging criteria, age and the medical history as well as laboratory data play an important role in establishing the correct diagnosis.

Kindliche Lebertumoren sind relativ selten (1 % kindlicher Tumoren), aber nicht unbedeutend [11]. Manche fallen durch Palpationsbefunde auf, viele sind aber Zufallsbefunde, die meist sonographisch in Erscheinung treten. Die Herausforderung besteht darin, die diversen Leberraumforderungen zu charakterisieren und benigne von malignen Veränderungen zu differenzieren. Hierbei spielt die Bildgebung neben biochemischen und Laborparametern eine wesentliche Rolle [19].

Wichtigste Bildgebungsmethode ist primär der Ultraschall (US), dessen Aussagekraft hoch ist, nicht nur bei der Detektion, sondern auch bei der Differenzierung und Klassifikation von Leberraumforderungen [17].

Ergänzt wird der Ultraschall vor allem durch die Magnetresonanztomographie (MRT), gelegentlich auch durch die Computertomographie (CT) – Letztere insbesondere im Rahmen des Stagings maligner Tumoren mit Lungenmetastasen oder zur Detektion kleinerer Verkalkungen. Bei der MRT sind gegatete oder atemgetriggerte Aufnahmen (eventuell in 3‑D-Technik und relativ enger Schichtdicke sowie multiplanarer isotroper Rekonstruktion) mit und ohne Fettsättigung, mit und ohne Kontrastmittel (KM; dynamisch – zur Beurteilung der KM-Dynamik) empfehlenswert und etabliert, wobei die Diffusionsbildgebung wesentliche Zusatzinformationen liefert und im Einzelfall auch leberspezifische KM hilfreich sein können (auch wenn diese im Kindesalter „off-label“ zu verwenden sind; [15]). Allerdings ist zu beachten, dass insbesondere im frühen Kindesalter die MRT meist nur in tiefer Sedierung oder Narkose durchgeführt werden kann [19].

## Rolle der radiologischen Methoden in der Abklärung von Lebertumoren im Kindesalter

Die Abklärung fokaler Leberläsionen des Kindesalters erfolgt primär mit US, weiterführend mittels MRT [2, 15]. Die CT eignet sich zwar prinzipiell zur Beurteilung, wird aber aufgrund der hohen Strahlenexposition selten eingesetzt [19]. Eine untergeordnete Rolle spielen Röntgen und Durchleuchtung, welche im Folgenden auch nicht weiter besprochen werden.

### Sonographie

Der moderne US ist die wichtigste Methode zur Beantwortung der meisten Fragestellungen rund um fokale Leberläsionen im Kindesalter [7, 19]. Die Vorteile sind die breite Verfügbarkeit, eine hervorragende Detailauflösung und die Beurteilbarkeit der Vaskularisation durch (Farb‑)Dopplermethoden; zusätzlich kann die intravenöse Gabe von US-Kontrastverstärkern hilfreich sein [3, 4]. In der Hand des erfahrenen Anwenders ist der US eine treffsichere Methode, wobei situationsbedingt verschiedene, insbesondere auch hochauflösende Linearschallköpfe erforderlich sind [17]. Die Sonoelastographie ist bislang insbesondere bei Kindern nicht etabliert.

#### Kontrastverstärkte Sonographie – „CEUS“

Ultraschallkontrastverstärker, auch US-KM genannt, spielen seit Jahren bei Erwachsenen eine wichtige Rolle bei der Detektion und Charakterisierung von Leberläsionen; ihre mit der CT vergleichbare diagnostische Zuverlässigkeit und das nebenwirkungsarmes Risikoprofil sind gut erforscht und dokumentiert. Sie erlauben die Beurteilung der KM-Dynamik und verbessern somit die Detektion und Charakterisierung von Leberläsionen wesentlich, auch wenn bei Kindern in Europa die intravenöse Gabe von US-KM „off-label“ ist. Zahlreiche Berichte zeigen die einfache, sichere und zuverlässige Anwendung auch bei Kindern, sodass die Food and Drug Administration (FDA) in den USA ein US-KM für die intravenöse Anwendung zur Beurteilung der kindlichen Leber zugelassen hat – insbesondere unter dem Aspekt, damit eine Alternative zur strahlenbelastenden CT zu etablieren, die sonst oft notwendig wäre und ggf. eine tiefe Sedierung oder gar Narkose erfordert. Voraussetzungen sind ein adäquates Gerät mit entsprechender, KM-tauglicher Schallkopfpalette, die Möglichkeit der Speicherung längerer Cine-loop-Clips (um die Anflutung und das Auswaschen des KM analysieren und dokumentieren zu können), ein „informed consent“ zur Kontrastmittelinjektion und entsprechendes Equipment vor Ort, um – falls erforderlich – allergische Reaktionen fachgerecht behandeln und monitorisieren zu können – obwohl diese sehr selten vorkommen. Ähnlich wie in der CT oder MRT ist ein frühes und kräftiges Enhancement mit einem raschen „wash-out“ malignitätsverdächtig; auch andere aus der CT und MRT bekannte Zeichen, wie z. B. das Irisblendenphänomen bei Hämangiomen sind im CEUS („contrast-enhanced ultrasonography“) in analoger Weise zu beobachten und können so rasch eine zuverlässige Diagnose ermöglichen [4].

### Magnetresonanztomographie

Die MRT eignet sich hervorragend zur Abklärung fokaler Leberläsionen, da sie zuverlässig zwischen Fett, Flüssigkeit und Weichteilen unterscheiden kann und dynamische KM-Sequenzen ermöglicht. Durch die Analyse der KM-Anflutung und -Ausschwemmung sowie dem Vergleich mit umgebendem Lebergewebe gelingt meist auch die Klassifizierung umschriebener Leberläsionen [15].

#### MRT-Kontrastmittel

Gadolinium (Gd) ist eine prinzipiell toxische Seltene Erde, welche bei Temperaturen ab 20 °C paramagnetische Eigenschaften hat. In der MRT kann es dennoch als intravenöses KM verwendet werden, indem es als Chelat appliziert wird, dessen Toxizität äußerst gering ist und das renal (und teilweise auch hepatisch) rasch eliminiert wird. Man unterscheidet zwischen extrazellulären und hepatobiliären KM sowie zwischen linearen und makrozyklischen Verbindungen. Da nach Applikation linearer Substanzen infolge einer, wenn auch geringen, Freisetzung in verschiedenen Geweben Ablagerungen elementaren Gadoliniums beobachtet wurden, sind in Europa bis auf Ausnahmen nur makrozyklische Gd-KM bei Kindern zugelassen, bei denen eine Freisetzung während der Verweilzeit im Körper praktisch nicht erfolgt. Die Ausnahmen bilden Gadoxetat und Gadobenat, die als leberspezifische KM von funktionstüchtigen Hepatozyten angereichert und zu unterschiedlich großem Anteil über die Galle ausgeschieden und daher in der Spätphase untersucht werden [23]. Da keine leberspezifischen, makrozyklischen KM verfügbar sind, muss zwangsläufig auf lineare KM zurückgegriffen werden, aber nur bei strikter Indikationsstellung. Eine Verwendung im Kindesalter erfolgt „off-label“, und in der Tat sind deren tatsächlichen Indikationen im Kindesalter sehr selten [15].

#### MRT-Technik und Protokolle

Die MRT fokaler Leberläsionen umfasst üblicherweise neben T2, T2FS, T1 „in/out of phase“ und Diffusion auch eine dynamische KM-Sequenz, welche als schnelle, T1-gewichtete fettgesättigte Gradientenechosequenz die KM-Verteilung gut zeigen kann. Die genaue Analyse der KM-Anflutung und -Ausschwemmung sowie der Vergleich mit umgebendem gesunden Lebergewebe erlaubt meist die Klassifizierung umschriebener Leberläsionen (Tab. 1; [15]).

**Table 1:** 

Sequenz	Schnittführung	Kommentar
T2	Axial oder koronal	–
T2 FS	Axial oder koronal	–
Diffusion	Axial	–
T1 in/out of phase	Koronal	Nativ, Beurteilung etwaiger Fettanteile
T1 GRE FS (4D) dynamisch	Axial oder koronal, evtl. mit Subtraktionen	Schnelle GRE-Sequenz mit FS, alle paar Sekunden wiederholt über ca. 4 min, Kontrastmittelkinetik und Gefäße gut beurteilbar
T1 TSE FS	Axial, koronal und/oder sagittal	In den beiden Ebenen
Evtl. leberspezifisches KM („off-label“)	–	Zur besseren Differenzierung zwischen normalem bzw. pathologischem Lebergewebe
Evtl. MRCP	–	Gallenwegdarstellung

*FS* „fat saturation“ (Fettunterdrückung), *GRE* Gradientenecho, *MRCP* MR-Cholangiopankreatikographie

### Computertomographie

Die CT spielt in der Abklärung fokaler Leberläsionen im Kindesalter keine große Rolle. Zwar kann sie anatomische Gegebenheiten mit hoher Genauigkeit auflösen, jedoch steht die hohe Strahlendosis der meist mehrphasig notwendigen Untersuchung einer breiteren Verwendung bei Kindern entgegen [14]. Dennoch kann eine CT in manchen Fällen sinnvoll sein, z. B. bei der präoperativen Darstellung des exakten Gefäßsitus, insbesondere, wenn in der MRT die Bildqualität eingeschränkt ist. Wenn eine CT notwendig ist, muss ggf. auf ausreichende Sedierung und immer auf die Anwendung kindes- und alters-/gewichtsadaptierter CT- und KM-Protokolle geachtet werden.

## Übersicht über Leberraumforderungen im Kindesalter

Die Leberraumforderungen variieren mit dem Alter: Unter 3 Jahren kommen vorwiegend vaskuläre Neoplasien, das Hepatoblastom, das Teratom, der rhabdoide Tumor und mesenchymale Hamartome vor. Neoplasien der Gallenwege wie das Rhabdomyosarkom (schon in frühem Kindesalter) oder Cholangiokarzinom (später auftretend) sind extrem selten und manifestieren sich klinisch zumeist durch Cholestase mit Ikterus. Des Weiteren anzuführen sind Hamartome und Leberzysten, deren Differenzialdiagnose schwierig sein kann [3, 11, 13].

Im späteren Alter können verschiedene Typen von hepatozellulären Karzinomen, aber auch Adenome oder eine fokale noduläre Hyperplasie (FNH) auftreten, insbesondere bei vorbestehender Zirrhose oder sonstigen Grunderkrankungen mit Leberbeteiligung. Lebermetastasen sind deutlich seltener als bei Erwachsenen und werden typischerweise beim Neuroblastom und bei Wilms- oder Keimbahntumoren beobachtet [3, 13].

Sonstige raumfordernde oder pseudotumoröse, differenzialdiagnostisch wichtige Veränderungen der Leber sind fokale Steatose/Non-Steatose, zystische Veränderungen (z. B. bei intrahepatischen Gallenwegs- oder Choledochuszysten, posttraumatische Zysten, zystische Systemerkrankungen, oder auch Echinococcus-Zysten) sowie Hämangiome oder Angiomyolipome. Auch fokale entzündliche Veränderungen wie ein entzündlicher Pseudotumor, (Pilz‑)Abszesse unterschiedlicher Größe oder auch regionäre Infiltrate bei systemischen Erkrankungen wie Leukämie können schwer von primären Leberraumforderungen zu unterscheiden sein (Tab. 2 und 3; [3, 10, 13, 14]).

**Table 2:** 

Benigne	Maligne	Tumor-like
Adenom	Cholangiokarzinom (extrem selten, später auftretend)	Infektion/Abszesse, Pseudotumor (Pilze, Bakterien, Parasiten z. B. Echinokokkose)
Fokale noduläre Hyperplasie (FNH)	Hämangiosarkom/epitheloides Hämangioendotheliom	Regeneratknoten und Narben
Hämangiom kongenital – RICH oder NICH, infantile oder adulte Form, Hämangiomatose = vaskuläre Malformationen	Hepatoblastom (meist unter 3 Jahren)	Steatose und Non-Steatose
Angiomyolipom (fast nur bei tuberöser Sklerose)	Hepatozelluläres Karzinom (verschiedene Typen, später auftretend, meist bei Lebervorerkrankung z. B. Zirrhose)	Zysten (verschiedener Ätiologie)
Mesenchymale Hamartome (meist unter 3 Jahren) und benigne Gallengangtumoren (Biliäres Zystadenom, papilläres Adenom, Gallenganghamartom)	Metastasen (z. B. Neuroblastom, Wilms- oder Keimbahntumoren) und Infiltrate bei malignen Systemerkrankungen (z. B. Leukämie …)	
Vaskuläre Neoplasien (meist unter 3 Jahren) – z. B. infantiles Hämangioendotheliom	Rhabdoider Tumor, Rahbdomyosarkom der Gallenwege (meist unter 3 Jahren)	
	Teratom (meist unter 3 Jahren)	

*RICH* „rapid involuting congenital hemangioma“, *NICH* „non-involuting congenital hemangioma“

**Table 3:** 

Entität	Merkmal	Arterielle Phase	Portalvenöse Phase	Spätphase
**Fehlende Zirrhose**				
*Adultes Hämangiom*	Typisch	Periphere noduläre Anreicherung	Teilweise/vollständige Kontrastierung	Vollständige Kontrastierung
	Zusätzlich	Kleine Läsionen: sofortige komplette Anreicherung	Zentripetale Füllung	Fehlende Anreicherung
*Infantiles Hämangiom (NICH/RICH)* *Hämangioendotheliom*	Typisch	Periphere Anreicherung	Partielle Anreicherung	Inkomplette Kontrastierung
	Zusätzlich	Frühe drainierende Vene. Kleine Läsionen: sofortige komplette Anreicherung	Zentripetale Kontrastierung	Regionen mit fehlender Anreicherung
*Fokale noduläre Hyperplasie (FNH)*	Typisch	Anreicherung vom Zentrum aus	Mehranreicherung	Isointens zur übrigen Leber
	Zusätzlich	Komplett, frühe Radspeichenarterien, Feeder-Arterie	Zentrale Narbe ohne KM-Anreicherung	Zentrale Narbe ohne KM-Anreicherung
*Andere kindliche Lebertumoren (variable Merkmale abhängig von der Größe)*	Typisch	Hyperkontrastierung	Isokontrastierung	Iso‑/Hypokontrastierung
	Zusätzlich	Regionen fehlender Anreicherung	Hypo- und hyperkontrastierte Areale	Nicht- und hypokontrastierte Anteile
*Hepatozelluläres Adenom*	Typisch	Komplette Mehranreicherung	Isointens zum übrigen Lebergewebe	Isointens zum übrigen Lebergewebe
	Zusätzlich	Regionen fehlender Anreicherung	Hypo- und hyperkontrastierte Areale	Nicht- und hypokontrastierte Anteile
*Fokale Steatose*	Typisch	Isointens	Isointens	Isointens
*Fokale Non-Steatose*	Typisch	Isointens	Isointens	Isointens
*Abszess*	Typisch	Periphere KM-Anreicherung	Hyper-/isoenhancing rim	Hypoenhancing rim
	Zusätzlich	Fehlende zentrale KM-Anreicherung, KM-aufnehmende Septen, Mehranreichernder Anteil	Fehlende zentrale KM-Anreicherung, hypointenser Randsaum, anreichernde Septen	Fehlende zentrale KM-Anreicherung
*Simple Zyste*	Typisch	Keine KM-Anreicherung	Keine KM-Anreicherung	Keine KM-Anreicherung
**Zirrhose**				
*Regeneratknoten*	Typisch	Nicht‑/Isointens	Isointens	Isointens
	Zusätzlich	Verminderte Anreichung bei dysplastischer Transformation	–	–
*Hepatozelluläres Karzinom*	Typisch	Hyperintens	Wash-out	Hypointens
	Zusätzlich	Kontrastmittelkinetik abhängig von Größe und Differenzierung des HCC	–	–

*KM *Kontrastmittel,* HCC *hepatozelluläres Karzinom*, RICH* „rapid involuting congenital hemangioma“, *NICH* „non-involuting congenital hemangioma“

### Benigne Lebertumoren

#### Hämangiom

Bei kleinen Kindern manifestieren sich Hämangiome anders als bei Erwachsenen. Angeborene Hämangiome („congenital haemangioma“, CH) involutieren entweder rasch (RICH = „rapid involuting CH“) oder nicht (NICH = „non-involuting CH“), was sich erst aus dem Verlauf sicher differenzieren lässt [6, 7]. Nach der neuesten Klassifikation werden diese als vaskuläre Malformationen eingestuft [15]. Meist sind sie echoarm und können zunächst vorübergehend an Größe zunehmen. Sonographisch zeigen sie keine sonstigen typischen Features, außer evtl. einem großen zuführenden Gefäß (wenn groß und mit hohem Shuntfluss) mit Niederwiderstandsflussprofil im Dopplersonogramm; der CEUS zeigt jedoch die hämangiomtypische KM-Dynamik im Sinne einer zunächst peripheren nodulären Anreicherung und nachfolgend zentripetaler Füllung, wie sie auch in der MRT beobachtet werden kann [3, 27].

Bei ausgeprägter, multifokaler oder diffuser Manifestation spricht man von Hämangiomatose der Leber, die oft schwer vom infantilen Hämangioendotheliom zu differenzieren ist. Diese können durch das große Shuntvolumen zu schwerer systemischer kardiovaskulärer Beeinträchtigung führen [3, 10, 14].

Das *infantile Hämangiom* ist bei der Geburt noch nicht vorhanden und entwickelt sich postnatal. Es ist selten, wird bevorzugt bei älteren Mädchen in der Leberperipherie subkapsulär gefunden und entspricht sonographisch dem typischen echoreichen, glatt begrenzten Bild des adulten Hämangioms. Bei großen (*Riesen-*)Hämangiomen (Durchmesser > 5 cm) kommt es häufig zu zentralen Hämorrhagien, Nekrose und Thrombose, so dass die diagnostische Einordnung anhand der Bildgebung schwierig sein kann, insbesondere, wenn eine sonstige Grunderkrankung vorliegt. Sie sind bei Kindern selten, und das typische Irisblendenphänomen im CEUS ermöglicht in der Regel die Diagnose, so dass keine zusätzliche MRT erforderlich ist [10, 13, 20].

Das typischerweise später, in etwa peripubertär entstehende *adulte Hämangiom* (mit entsprechend typischem sonomorphologischem Aspekt) zeigt keine Unterschiede zum Erwachsenen, muss aber von ähnlichen Tumoren, wie Angiomyofibromen (z. B. bei einer tuberösen Sklerose), unterschieden werden [14].

#### Leberadenom und nodulär regenerative Hyperplasie

Adenome sind in der kindlichen Leber selten; meist betreffen sie ältere Kinder mit Vorerkrankungen (d. h. mit Lebererkrankungen, z. B. bei Glykogenose, Thyrosinose, Alpha-1-Antitrypsinmangel, nach Chemotherapie). Ihr Erscheinungsbild ist unspezifisch – im US sind sie meist als echoarme Rundherde zu sehen, evtl. mit Pseudokapsel. In der MRT ist ihre Signalintensität in T1w variabel; in T2w sieht man meist diskret hyperintensen Rundherde. Im CEUS beobachtet man eine starke frühe, oft peripher betonte und zentripetale Anflutung, gefolgt von Isointensität zur umliegenden (gesunden) Leber; auch Regionen ohne KM-Aufnahme (Fetteinlagerungen, Einblutung) kommen vor. Schwierigkeiten bereitet die Abgrenzung gegen z. B. Lebermetastasen, Regeneratknoten (nodulär-regenerative Hyperplasie in z. B. zirrhotischen Lebern, mit oft weniger und meist fehlendem früharteriellem Hyperenhancement) oder einer FNH, sogar bei Einsatz von US-KM [15].

#### Fokale noduläre Hyperplasie

Nach dem Hämangiom ist die FNH die zweithäufigste gutartige Leberläsion. Erwachsene sind häufiger betroffen als Kinder, Mädchen öfter als Jungen. Das charakteristische Merkmal dieser meist scharf umschriebenen nodulären Raumforderung ist die zentrale Narbe mit radiären fibrösen Septen; die Morphologie kann aber untypisch sein. Zwar ist die zentrale Narbe typisch und spezifisch für die FNH, aber nur in weniger als der Hälfte der Fälle ist sie nachzuweisen. Ein weiteres typisches Zeichen einer FNH ist eine prominente zentrale Arterie mit radspeichenartiger Kontrastierung [24]. Radiologisch eignen sich insbesondere der US und MRT, prinzipiell auch die CT (*Cave:* Strahlendosis) – jeweils mit intravenöser KM-Gabe. Im US kann im B‑Bild die FNH von echogleicher Echostruktur und demzufolge schlecht sichtbar sein. In der arteriellen Phase zeigt sich eine kräftigere Kontrastierung als das benachbarte Lebergewebe, anschließend füllt sich die Läsion abgesehen von einer etwaigen Narbe zentrifugal. Im Gegensatz zum Adenom persistiert die Kontrastierung in den späteren Phasen. In der MRT lässt sich bei unklaren Fällen eine FNH mithilfe eines leberspezifischen Gd-haltigen KM anhand einer diskret verstärkten Anreicherung in der Spätphase mit hoher Genauigkeit diagnostizieren [20]. Als wichtige Differenzialdiagnosen sind das Leberadenom und das Hämangiom zu nennen, welche aber beide typischerweise keine Spätkontrastierung aufweisen [3, 15, 20].

#### Mesenchymales Hamartom

**Figure 1:**
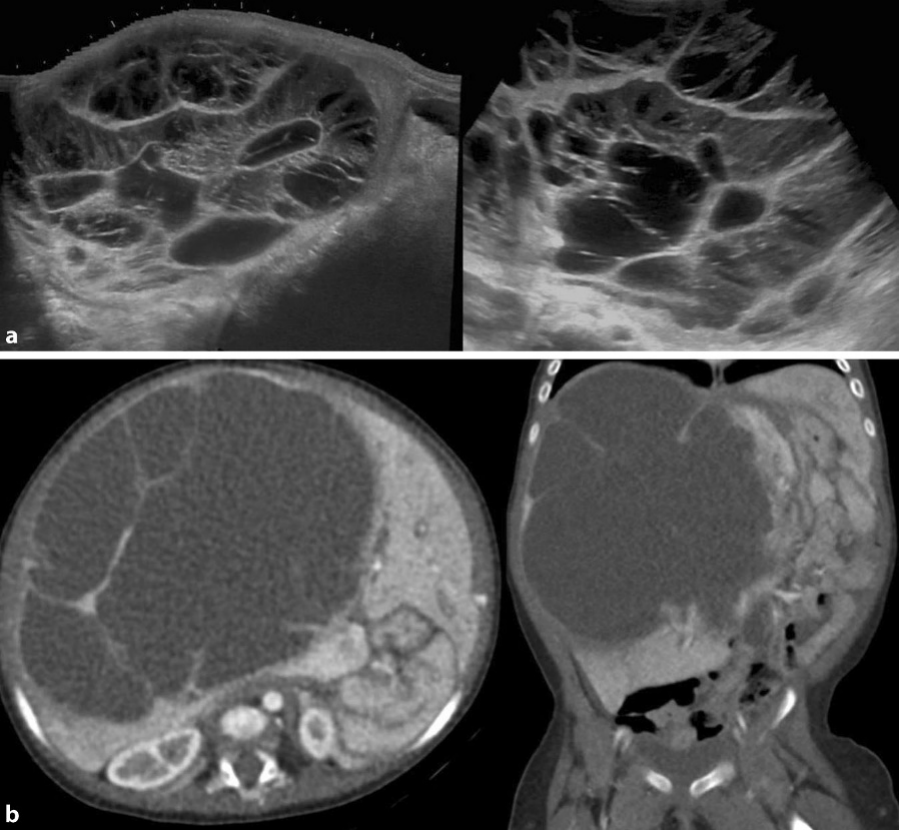

Leberhamartome sind meist im rechten Leberlappen gelegen und können groß werden, allerdings ist keine Entartungstendenz beschrieben. Das Erscheinungsbild variiert stark von fast homogen solide bis zu großteils zystisch – mit oder ohne Septen (Abb. 1). Die Diagnose ist nur histologisch sicher zu verifizieren – weder der US noch der CEUS oder die MRT erlaubt eine sichere bildgebende Differenzierung von anderen Entitäten [3, 14].

#### Lipom

Das extrem seltene isolierte Leberlipom ist ein benigner mesenchymaler Tumor ohne Entartungsrisiko und ähnelt sonographisch etwas einer auffallend gut begrenzten fokalen Steatose oder einem Angiomyolipom. Der Beweis des Fettgehalts kann mittels MRT unter Verwendung von Fettunterdrückung und In-/out-of-phase-Sequenzen gelingen [3, 4].

#### Angiomyolipom

**Figure 2:**
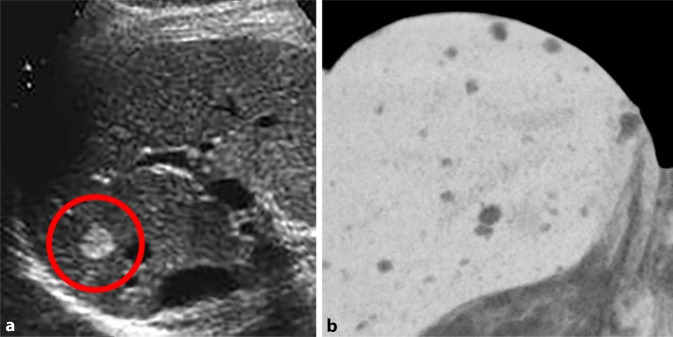

Angiomyolipome sind seltene hamartöse Lebertumoren und bestehen aus Blutgefäßen, glatter Muskulatur und Fettgewebe; sie werden fast nur bei der tuberösen Sklerose gesehen, wenngleich seltener als in den Nieren. Im US stellen sie sich meist als echoreich-kugelige Areale dar (Abb. 2). Eine weiterführende Abklärung ist in der Regel nicht notwendig [3, 4, 18].

#### Gallengangtumoren

Cholangiozelluläre Karzinome kommen auch bei Jugendlichen vor, sind aber äußerst selten. Etwas häufiger werden benigne Gallengangtumoren wie das biliäre Zystadenom, papilläre Adenom oder Gallenganghamartom gesehen. Sie äußern sich durch eine Cholestase und können mittels Sonographie und MRT beurteilt werden [5].

### Maligne Lebertumoren

Bösartige Lebertumoren im Kindesalter sind selten. Die wichtigsten Entitäten sind hier das Hepatoblastom und das hepatozelluläre Karzinom (HCC). Sarkome und vaskuläre Malignome sind selten [28, 29].

#### PRETEXT-Klassifikation

**Figure 3:**
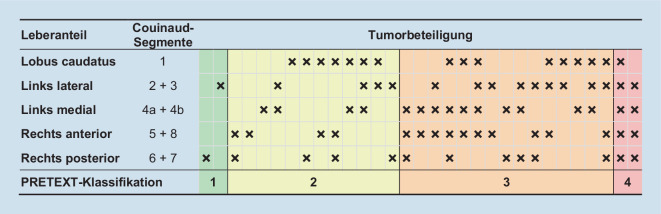

Das PRETEXT-Klassifikationsschema („pre-treatment extent of tumor“) kindlicher Lebertumoren, vorgeschlagen von der *International Childhood Liver Tumors Strategy Group*, versucht die Vergleichbarkeit von Staging und Risikoabschätzung zu verbessern [25]. Sie wurde zunächst für Hepatoblastome definiert, kann aber in der aktuellen Fassung für alle kindlichen Lebertumoren angewendet werden. PRETEXT baut auf den Couinaud-Segmenten der Leber auf, welche in 4 Abschnitte eingeteilt wird. Daraus leitet sich die PRETEXT-Nummer (1 bis 4) ab (Abb. 3). Zusätzlich werden Tumorinfiltration und -metastasierung mittels Zusatzkategorien, repräsentiert durch Großbuchstaben, bewertet. Wie alle Klassifikationen gibt es eine gewisse Unschärfe und Subjektivität in der Einteilung, weshalb diese meist in Zentren bzw. den Studienzentralen vorgenommen wird [25, 26, 30]. Neben PRETEXT gibt es auch Bestrebungen, die LI-RADS-Klassifikation brauchbar für Kinder zu adaptieren [12, 21, 22].

#### Hepatoblastom

**Figure 4:**
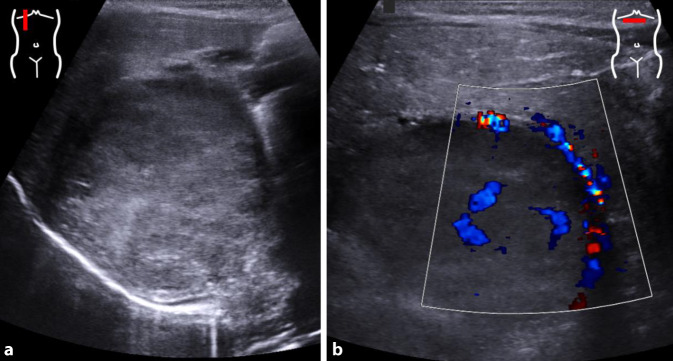

Hepatoblastome treten meistens im Säuglings- oder Kleinkindesalter auf, mit unspezifischen Symptomen. Ein bekannter Risikofaktor ist die Frühgeburtlichkeit. Laborchemisch kann ein erhöhter Alpha-1-Fetoprotein-Wert hinweisend sein. Der initiale US liefert oft den ersten Nachweis einer üblicherweise großen inhomogen und relativ umschriebenen, meist singulären Leberraumforderung ([1, 13, 16]; Abb. 4). Verkalkungen sind möglich, dann aber relativ typisch. Die MRT wird hauptsächlich zur Beurteilung der genauen Ausdehnung und einer etwaigen Gefäßbeteiligung sowie zur Detektion pathologischer Lymphknoten eingesetzt und zeigt in der Regel eine Raumforderung mit vermehrtem Flüssigkeitssignal und heterogener KM-Anreicherung [15, 18].

#### Hepatozelluläres Karzinom

Der zweithäufigste maligne Lebertumor des Kindesalters ist das hepatozelluläre Karzinom. Typischerweise tritt das kindliche HCC ab dem 5. Lebensjahr auf [1, 11]. Etwa 20 % der Fälle entfallen dabei auf die fibrolamelläre HCC-Variante, welche bevorzugt Jugendliche betrifft. Chronische Leberinfektion (Hepatitis B oder C) sowie Stoffwechselerkrankungen können das Auftreten begünstigen [9]. Laborchemisch ist in der Mehrheit der Fälle der Alpha-1-Fetoprotein-Spiegel erhöht. Der US zeigt meist einen heterogenen Leberprozess unterschiedlicher Ausdehnung und mit verstärkter Gefäßzeichnung [28]. Eine Infiltration der Pfortader oder der Lebervenen ist möglich. Dynamische Kontrastmitteluntersuchungen (CEUS, MRT) zeigen eine vermehrte arterielle Kontrastierung mit typischem „wash-out“ in der portalvenösen und verzögerten Phase [15].

#### Lebersarkome

Sarkome der Leber sind selten, aber nach dem Hepatoblastom und dem HCC die dritthäufigste maligne Entität. Man unterscheidet undifferenzierte embryonale Sarkome, Rhabdomyosarkome und Angiosarkome. Meist präsentieren sie sich als große singuläre hepatische Raumforderung, welche Nekroseareale (sonographisch stark hypoechogen bzw. echofrei, fehlende KM-Aufnahme in CEUS oder MRT) aufweisen können und auch komplex-zystisch imponieren. Die KM-Anreicherung ist unspezifisch und sehr variabel. Die Histologie ist zur Diagnosefindung unerlässlich [1, 15].

#### Epitheloides Hämangioendotheliom

**Figure 5:**
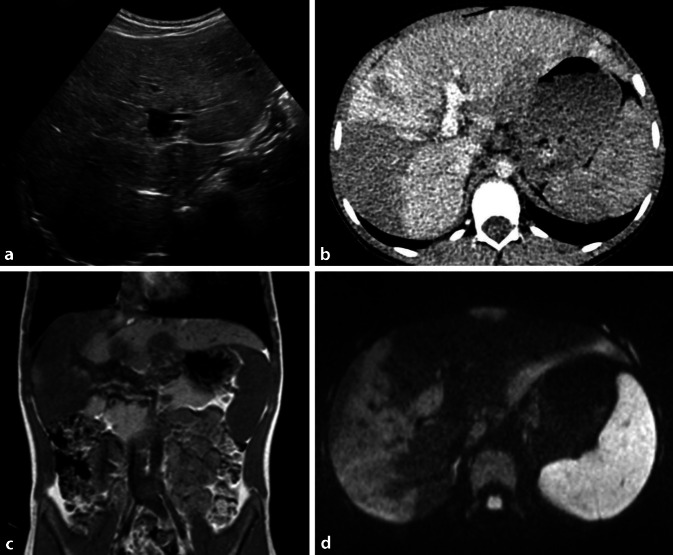

Das niedrig bis moderat maligne epitheloide Hämangioendotheliom der Leber ist eine Rarität, die sogar bei Neugeborenen auftreten kann ([6]; Abb. 5). Dieser vaskuläre Tumor befällt neben der Leber auch andere Organe, beispielsweise die Lungen. Er wächst in die peripheren Gefäße vor und okkludiert sie, wodurch, im Gegensatz beispielsweise zu einem Angiosarkom, eine verminderte KM-Anreicherung zu beobachten ist. Radiologisch findet man multiple kleinknotige oder flächige, häufig konfluierende oder multifokale Läsionen, die sich in der CT typischerweise hypodens, im US als echoarm, und in der MRT mit hohem Flüssigkeitssignal präsentieren [17]. Manchmal fällt die Unterscheidung zwischen normalem und pathologischem Lebergewebe schwer, auch die Tumorgrenzen sind oft nicht klar. In solchen Fällen kann die Gabe eines leberspezifischen MR-KM zur Unterscheidung zwischen normalem und pathologischem Lebergewebe erwogen werden [2].

### Lebermetastasen

Lebermetastasen können auch bei Kindern im Rahmen einer Vielzahl von Malignomen auftreten. Die Bildgebung ist daher heterogen. Mehrheitlich stellen sie sich als multiple echoarme, hypodense bzw. T2-hyperintense Läsionen dar [5, 8].

### Tumor-like lesions

#### (Kongenitale) Leberzyste(n)

Leberzysten können angeboren oder erworben sein (z. B. posttraumatisch, als Residuen eines Abszesses). Insbesondere bei größeren, in der Bildgebung komplex aufgebaute Zysten und Zystenkonglomeraten ist weder mittels US noch CEUS oder MRT eine sichere Differenzierung von anderen Entitäten möglich (z. B. Echinococcus-Zyste Typ IV/V nach Gharbi, zystisches Hamartom oder Teratom). Des Weiteren sind Leberzysten im Rahmen zystischer Systemerkrankungen (d. h. Ziliopathien) zu beobachten. Auch zystisch erweiterte Gallenwege/Gallenwegresiduen (z. B. „central cyst sign“ bei Gallengangatresie) oder ektatisch-aneurysmatisch erweiterte Gefäße können diagnostische Probleme bereiten. Unkomplizierte Zysten (sonographisch einfach, zartwandig, ohne Inhalt) werden meist nur mit dem US kontrolliert, bei Unklarheiten und komplizierten Zysten (z. B. posttraumatisch) wird evtl. der Zysteninhalt unter US-Sicht punktiert und analysiert (z. B. epitheliale Zyste, Differenzialdiagnose Biliom …) – nur sehr selten ist eine MRT indiziert [3, 4, 13].

#### Fokale Steatose/Non-Steatose

**Figure 6:**
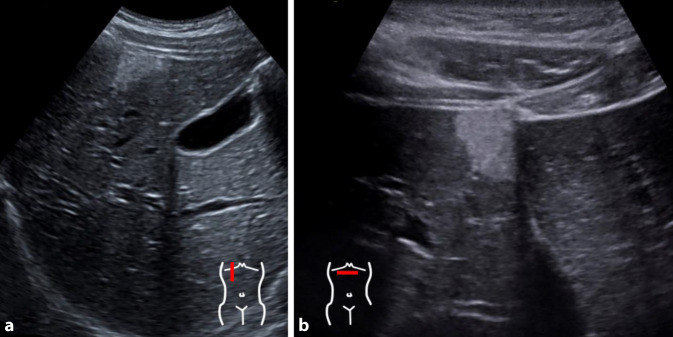

Fokale Steatoseareale kommen auch im Kindesalter vor, wenn auch seltener als bei Erwachsenen (Abb. 6). Die typische Lokalisation befindet sich im Segment IV des linken Leberlappens und angrenzend an das Gallenblasenbett. Durch den erhöhten Fettanteil stellen sich fokale Steatoseareale im US üblicherweise echoreich dar, während die Non-Steatose als echoarme Bezirke in einer echoreich-verfetteten Leber imponieren. Als wichtigste Differenzialdiagnose kann bei fokaler Steatose das Leberhämangiom, das ebenfalls häufig Fettanteile aufweist, oder bei Non-Steatose das Adenom angesehen werden [29]. Eine weiterführende MRT ist im Regelfall nicht indiziert.

#### Leberabszess

Leberabszesse sind singuläre oder multiple infektiöse Einschmelzungen des Leberparenchyms. Sie können durch Bakterien, Pilze oder Parasiten ausgelöst werden. In der Bildgebung findet man mäßig scharf berandete Herd(e) mit randständig betonter, membran- oder kapselförmiger und zentral fehlender KM-Anreicherung. Gaseinschlüsse sind möglich und relativ charakteristisch. Die zentrale Nekrosehöhle kann in der MRT eine ADC-Absenkung zeigen. Ein *umgekehrtes Zielscheiben-Phänomen* mit echoarmem, T2-signalreichem oder CT-graphisch hypodensem Zentrum und einem dickwandigen, KM-aufnehmenden Randsaum ist hinweisend für einen Leberabszess [29].

#### Echinokokkose

**Figure 7:**
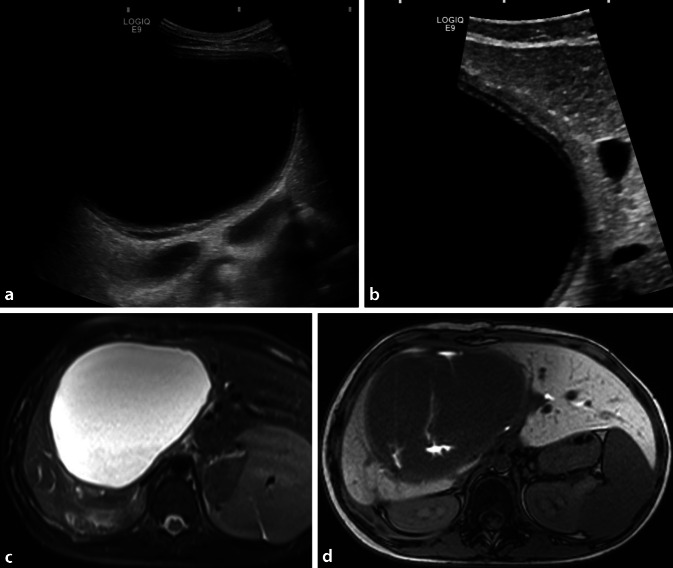

In den letzten Jahren sehen wir vermehrt Echinokokkose-Fälle, sodass bei einer zystischen Leberraumforderung auch hieran gedacht werden muss (Abb. 7). Der Anstieg ist primär durch Phänomene wie Globalisierung und Migration zurückzuführen. Die meist großen Zysten (die u. A. auch in anderen Organen, der Bauch- und Thoraxhöhle sowie zerebral vorkommen können) weisen eine verhältnismäßig charakteristische Morphologie auf, können aber ein recht unterschiedliches und manchmal irreleitendes Bild aufweisen (Gharbi-Klassifikation Typ I–V, Tab. 4). Meist ist eine geschichtet-dickwandige Zyste evtl. mit Tochterzysten sichtbar, wobei die innere Schicht kollabiert im Lumen liegt und bei älteren Zysten auch eine Wandverkalkung vorliegen kann, während ganz frische Zysten (Gharbi Typ I) nicht immer sicher von einer simplen Leberzyste zu unterscheiden sind. Bildgebung der Wahl sind der US und bei Unklarheit die MRT. Sollte die Verdachtsdiagnose einer Echinokokkose im Raum stehen, so ist es wichtig, den Verdacht ausreichend zu kommunizieren, da eine iatrogen herbeigeführte Zystenruptur (z. B. bei einer Punktion) lebensbedrohliche Komplikationen verursachen kann [17].

**Table 4:** 

Typ I	Echofreie kugelige Zyste ohne Inhalt (Differenzialdiagnose zu simpler Zyste schwierig/sonomorphologisch unmöglich), jüngstes Stadium
Typ II	Wie Typ I, aber mit doppelkonturierter Grenzschicht (quasi pathognomonisch)
Typ III	Wie Typ II, aber mit multiplen Tochterzysten innerhalb sowie außerhalb der Hauptzyste, evtl. partielles Detachment
Typ IV	Heterogene Läsion mit flüssigen und soliden Komponenten, sonomorphologisch nicht sicher von (malignen) Lebertumoren zu differenzieren
Typ V	Verkalkungen in der Zystenwand (ältestes Stadium)

## Fazit für die Praxis

- Lebertumoren im Kindesalter sind selten; das Wissen über deren Erscheinungsbild und eine adäquate diagnostische Aufarbeitung sind essenziell, wobei labor- und biochemische Parameter sowie das Alter des Kindes bzw. Vorerkrankungen wegweisend sein können.
- Die Verwendung moderner sonographischer Methoden ermöglicht eine sehr gute Detektion und Charakterisierung der diversen Leberläsionen durch hochauflösende Schallköpfe, (Farb‑)Dopplersonographie und CEUS, künftig möglicherweise auch der Sonoelastographie.
- Bei malignen Erkrankungen muss der US durch die MRT ergänzt werden.
- Die CT stellt mit Blick auf die Strahlenbelastung eine Reservemethode dar.
- Festgelegte Befundungs- und Stagingprotokolle (z. B. PRETEXT) sollten angewendet werden – hierfür ist die enge Zusammenarbeit mit den pädiatrischen Onkologen essenziell.
- Die Diagnostik und Behandlung bzw. das Management bei Verdacht auf einen kindlichen Lebertumor sollte an dezidierten (kinderradiologischen) Tumorzentren erfolgen.
